# Prevention and Treatment of Oral Complications in Hematologic Childhood Cancer Patients: An Update

**DOI:** 10.3390/children9040566

**Published:** 2022-04-15

**Authors:** Alba Ferrández-Pujante, Amparo Pérez-Silva, Clara Serna-Muñoz, José Luis Fuster-Soler, Ana Mª Galera-Miñarro, Inmaculada Cabello, Antonio J. Ortiz-Ruiz

**Affiliations:** 1 Department of Integral Paediatric Dentistry, Faculty of Medicine and Dentistry, University of Murcia, 30008 Murcia, Spain; alba_13_1991@hotmail.com (A.F.-P.); amparo.perez@um.es (A.P.-S.); claraserna@live.com (C.S.-M.); ajortiz@um.es (A.J.O.-R.); 2 Institute of Biomedical Research, IMIB, 30120 Murcia, Spain; josel.fuster@carm.es (J.L.F.-S.); anam.galera@carm.es (A.M.G.-M.); 3 Paediatric Oncology Section, Children’s University Hospital Virgen of Arrixaca, 30120 Murcia, Spain

**Keywords:** oral prophylaxis and oncology, oral hygiene and oncology, oral care in paediatric oncologic patients, dental management and oncology

## Abstract

Cancers have a highly negative impact on the quality of life of paediatric patients and require an individualised oral treatment program for the phases of the disease. The aim of this study was to update existing research on oral care in children diagnosed with cancer. We carried out a literature search (in English, Spanish and Portuguese) in the Pubmed, Cochrane Library, EBSCO, WOS, SciELO, Lilacs, ProQuest, and SCOPUS databases and the websites of hospitals that treat childhood cancers. We found 114 articles and two hospital protocols. After review, we describe the interventions necessary to maintain oral health in children with cancer, divided into: phase I, before initiation of cancer treatment (review of medical record and oral history, planning of preventive strategies and dental treatments); phase II, from initiation of chemo-radiotherapy to 30–45 days post-therapy (maintenance of oral hygiene, reinforcement of parent/patient education in oral care, prevention and treatment of complications derived from cancer treatment); phase III, from 1 year to lifetime (periodic check-ups, maintenance, and reinforcement of oral hygiene, dental treatments, symptomatic care of the effects of long-term cancer treatment). The use of standardised protocols can avoid or minimise oral cancer complications and the side effects of cancer therapies.

## 1. Introduction

### 1.1. Paediatric Oncology

#### 1.1.1. Epidemiology

The incidence of childhood cancer is highest in North America, parts of South and Central America, Europe, and Australia with an age-standardised incidence rate of ≥ 15.4 per 100,000 person-years for those 0–19 years of age [1]. Despite therapeutic advances and the fact that childhood cancer is a rare disease, it is the leading cause of death from disease until 14 years of age in high-income countries [2].

The most frequent types of cancer in children are leukaemia (25–35%), predominantly acute leukaemia (acute lymphoblastic leukaemia (ALL) (78%) and acute myeloblastic leukaemia (16%)), central nervous system tumours (22.6%), lymphomas (15.6%), and neuroblastomas or sympathetic nervous system tumours (7.8%) [3].

The prognosis of childhood cancer has improved dramatically in recent years, reaching a worldwide 5-year survival rate of 84% in children aged 0–14 years. The rate is similar in most European countries, including Spain (78%), Italy and the UK (82%), Germany (84%), Austria (85.9%), and the USA (83%), but is higher than in Eastern Europe (60–77%) [3].

#### 1.1.2. Clinical Signs and Symptoms

Initially, patients with leukaemia are asymptomatic but, as it progresses and bone marrow infiltration occurs, haematopoiesis fails and peripheral cytopenia occurs, leading to anaemic syndrome and B symptoms (fever without infection, weight loss, night sweats). Blastic infiltration of other organs results in hepatosplenomegaly, lymphadenopathy, bone pain, central nervous system infiltration, mediastinal mass due to thymus growth, testicular infiltration, and infiltration of the skin and gums, depending on the type of leukaemia [4]. In patients with ALL, oral manifestations include pallor of the mucous membranes, petechiae, gingival bleeding, oral ulcers, gingival hypertrophy, palpable lymph nodes, laryngeal pain, sore throat, mucositis, candidiasis, gingivitis, and periodontitis. Patients may develop infections and haemorrhages due to neutropenia, immunodeficiency, and thrombocytopenia [5,6].

Cancers in children have a higher potential for growth and development than in adults and both the disease and its treatment may seriously impair normal development [7].

#### 1.1.3. Treatment

Deep genetic sequencing has offered an unprecedented vision of the biology of cancer and has resulted in huge advances in the treatment of leukaemia. With the advent of precision medicine, directed therapy is becoming increasingly applicable, with new chemotherapeutic agents focused on cancer-specific biology, including biological drugs, targeted therapy, monoclonal antibodies, anti-angiogenics, and chimeric antigen T-cell receptor immunotherapy. Despite these advances, surgical resection, chemotherapy, and radiotherapy remain the main therapies for childhood cancer [8].

Leukaemia therapy depends on factors such as the disease type and subtype, the risk factors, and the patient’s age. In general, the treatment of choice is chemotherapy with or without coadjuvant treatments (radiotherapy, corticosteroids) and haematopoietic stem cell transplantation (HSCT) or bone marrow transplantation, which is generally carried out in acute cases and some chronic myeloid leukaemia cases [9].

Standard chemotherapy normally includes four well-defined therapeutic stages: induction therapy, preventive therapy or central nervous system prophylaxis, consolidation or intensification, and maintenance or continuation [6,10].

### 1.2. Oral Disease in Childhood Cancer

Oral complications may be due to the cancer itself or to the treatment received [10], and vary according to the age at diagnosis and the type of chemotherapy, the dose used, and the area of irradiation in the case of radiotherapy [11,12]. Oral complications are mostly associated with pre-existing factors (caries, gingivitis, and poor hygiene) that affect their initiation, increase, and persistence [7].

Childhood cancer patients may have many short- and long-term oral complications, which include specific oral tissue manifestations (alterations in the mucosa, salivary glands, muscle and bone, sensory alterations, alterations in teeth and gums) and non-specific oral tissue manifestations (oral bleeding, opportunistic infections, secondary tumours, post-transplant lymphoproliferative disorders, dental anomalies, and craniofacial alterations) (Table 1).

The problem with the existing guidelines for the management of prevention and treatment of oral complications in these childhood cancer patients is that they only establish measures for four well-defined therapeutic stages.

Therefore, it is necessary to establish a protocol that includes the prevention and treatment of possible oral complications in these patients throughout all the phases of treatment of their pathology.

Different tools are available to record the incidence of acute and chronic oral complications of oncology treatment and assess the effectiveness of interventions. Validated mucositis assessment tools in paediatric patients include the World Health Organization (WHO) functional scale, the eat and drink ability-based scale of the National Cancer Institute Common Toxicity Criteria [21], the Children’s International Mucositis Evaluation Scale (ChIMES) [22], the Oral Assessment Guide (OAG) [23], the modified Oral Assessment Guide (OAG) [24], the Oral Mucositis Assessment Scale [25] (and the Oral Mucositis Daily Questionnaire [26], and the Guidelines of the Children’s Oncology Group oral-dental panel [27]. Some of these scales not only quantify changes in the integrity of the oral cavity but may also be useful in the early identification of complications that require prompt therapy to reduce morbidity in immunosuppressed patients and decrease the severity of oral changes before, during, and after chemotherapy [28].

Successful management of oral complications, including systemic infections of oral origin, should begin with the oral examination, the introduction of comprehensive oral hygiene measures, and definitive dental interventions prior to initiation of cancer therapy. The incorporation of a paediatric dentist in the multidisciplinary team in childhood cancer, and the establishment of standardised protocols based on prevention, and not just the treatment of the complications is, accordingly, necessary in order to ensure successful cancer treatment and the best quality of life.

The aim of our study was to update existing knowledge on oral complications in children with cancer and the management after diagnosis.

## 2. Materials and Methods

### Search

We carried out a literature search (in English, Spanish and Portuguese) in Pubmed, Cochrane Library, EBSCO, WOS, SciELO, Lilacs, ProQuest, SCOPUS, and the websites of hospitals that treat childhood cancers.

The strategy search was: (“Dental Prophylaxis” OR “oral prophylaxis” OR “oral hygiene” OR “dental Care for Children” OR “oral care” OR “dental treatment”) AND (“pediatric cancer” OR “pediatric oncology” OR “childhood oncology” OR “childhood cancer” OR “childhood blood cancer” OR “pediatric hematologic neoplasms” OR “childhood hematologic neoplasms”) AND (“protocol” OR “guide” OR “guideline”), making the appropriate adaptations to the language required by the different databases.

The search yielded 114 scientific articles from the databases: Pubmed (12), Cochrane Library [clinical trial (7), reviews (2)], EBSCO (12), WOS (13), SciELO (3), Lilacs (3), ProQuest (11), SCOPUS (51). Of these, 28 were repeated in different databases (Supplementary File S1).

For this review, we have used 29 articles obtained from the main search strategy [13,14,21,23,24,26,27,28,29,30,31,32,33,34,35,36,37,38,39,40,41,42,43,44,45,46,47,48,49], articles obtained from searches of specific topics, two hospital protocols (Women’s and Children’s Hospital, North Adelaide, South Australia [15,18,50,51]; University Hospital Sant Joan de Déu, Barcelona, Spain [52]) and the Dental Management of Pediatric Patients Receiving Immunosuppressive Therapy and/or Radiation Therapy recommendations of the American Academy of Pediatric Dentistry [53].

The last revision was made in March 2022. We have not found any new protocols.

## 3. Results

### 3.1. Phase 1: From the Diagnosis of Cancer to Initiation of Chemo/Radiotherapy

The aims of phase 1 are:

- To identify and eliminate possible sources of infection and local irritants in the oral cavity in order not to delay cancer treatment or induce other complications.
- To educate patients/parents on the importance of following optimal oral care to minimise oral problems before, during, and after cancer treatment.
- To advise patients/parents about the possible short- and long-term side effects of cancer treatment in the oral cavity and craniofacial complex.

During this phase, oral lesions should be treated and dental rehabilitation carried out before initiating cancer therapy, although this is not always possible. Possible obstacles include:

- Urgent cancer treatment at diagnosis (as in cases of acute leukaemia) leaving no time to complete the necessary dental treatments.
- The risk of osteoradionecrosis if the patient has recently received or will soon receive radiation therapy of the head and neck, which is a contraindication for some treatments, such as tooth extractions [39].

In these cases, a deviation from the strategy will be necessary to adapt it to the specific situation of the patient, who will start directly in phase II (skipping phase I) and avoid invasive dental procedures.

### 3.2. Systematic Measures in Patients Newly Diagnosed with Cancer

1. Review of medical records and the proposed cancer protocol.
  - Determine current medical condition and underlying disease (type, stage, and prognosis) [11,54].
  - Determine the proposed cancer treatment protocol and its cycles (conditioning regimen, surgical resection, chemotherapy, radiotherapy, and transplant) [11,54].
  - Determine allergies, previous operations, and secondary medical illnesses [11].
  - Review and adjust, as far as possible, the medication (reduction/suppression of xerogenic drugs, or substitution by non-xerogenic drugs, need or not for bisphosphonates, etc.) [54].
  - Establish a multidisciplinary treatment plan based on the current haematological/systemic status, current oral situation, dates of cancer surgery, chemotherapy, radiotherapy, and transplantation, in order to initiate oral prevention and treatment strategy as soon as possible.
  - Review haematological status (complete blood count):
    (a) A permanent venous catheter (most patients with leukaemia have a central line with a permanent catheter inserted in the right atrium of the heart, both for the administration of prolonged chemotherapy and for nutritional and electrolyte supply) [6,7,54].
    (b) The American Heart Association indicates that antibiotic prophylaxis is not necessary for patients with permanent central venous catheters who undergo dental procedures except at the time heart devices are placed (to prevent postoperative infections) or in immunosuppressed patients [6,55,56].
      − Immunosuppressive status (total neutrophil count) [6,9]: >2000/mm^3^: antibiotic prophylaxis is not indicated.
      − 1000–2000/mm^3^ or if there is an infection or the status is doubtful: antibiotic prophylaxis is indicated.
      − <1000/mm^3^: postpone dental treatment and, in case of urgency, decide together with the medical team whether to carry out the dental treatment under antibiotic prophylaxis: this requires hospitalisation.
    (c) Coagulation status (total platelet count) [6,9]:
      − >75,000/mm^3^: does not require additional support to carry out the dental treatment.
      − A 40,000–75,000/mm^3^: platelet transfusion is required before and 24 h after dental treatment. If treatment involves bleeding, haemostatic measures such as local haemostatic agents, sutures, sterile gauze to compress the bleeding area and/or microfibrillar collagen sponges, will be required.
      − <40,000/mm^3^: dental treatment must be postponed and, in case of urgency, decide together with the medical team whether to perform the treatment in hospital, with platelet transfusion, bleeding control measures (sutures, sterile compression gauzes, microfibrillar collagen sponges, local haemostatic agents such as topical thrombin) and additional medication for bleeding control (aminocaproic acid, tranexamic acid).
2. Oral history
  - Record oral hygiene habits, dietary habits, history of trauma, exposure to fluoride in the form of fluoridated water/fluoridated salt or fluoride supplements (in pills, gel, or varnish).
  - Intraoral examination (must be performed immediately after diagnosis of cancer [57]: perform a thorough examination of the oral cavity, and record in odontogram (symptomatic teeth, dental treatments performed, dental treatments necessary, prostheses or fixed and removable orthodontic appliances, space maintainers, temporary teeth with mobility in the process of natural exfoliation, aphthae or ulcerative lesions, lesions in the oral mucosa and tongue, etc.) and in the periodontogram (gingivitis, periodontitis, pericoronaritis, gingival recession, periodontal abscess, dental mobility, periodontal pouch, furcation injuries, etc.).
  - Extraoral examination: perform a complete examination of the head and neck by palpation of submandibular ganglion chains, temporomandibular joint (TMJ), lips, etc., (note trigger points, pain on palpation of facial muscles, trismus, TMJ pain, herpes simplex, dry skin, etc.).
  - Complete the examination with a radiographic study based on panoramic and intraoral radiographs (bitewing and/or periapical), according to dental criteria and individual patient needs [6,54,55,58,59,60].
3. Planning of preventive strategies
  - General oral prophylaxis measures:
    (a) Teach correct brushing technique (Bass method) [29,49,61,62]. Brush teeth and tongue with the supervision and assistance of adults using a small hand-held manual nylon toothbrush of mild-medium hardness or an electric toothbrush (if the patient is trained), 2–3 times a day (regardless of haematological status) [6,36,52,55], for at least 2 min. Reinforcement and motivation of hygiene techniques at least every 3 months with plaque controls (erythrosine or Tri plaque). Let the toothbrush dry between brushings [50].
    (b) Use fluoride toothpaste (1000 ppm of fluoride in children aged <6 years: a smear between 0–3 years and a grain of rice in patients aged 3–6 years and ≥1450 ppm of fluoride in children aged >6 years (size of a pea). Spit when finished without subsequent rinsing [63]. Choose dentifrices with relatively neutral flavour [53].
    (c) Use dental floss/floss only in trained patients or under adult supervision, at least once a day (before sleep) before brushing teeth [61,64]. Dental arches may be used for greater ease.
    (d) Preventive application of fluorinated preparations and antimicrobial products (mouthwash, gel, or varnish):
      − Topical application of sodium fluoride, repeated every 3 months or less (according to individual need), as gel or varnish, depending on age: varnish in children aged < 6 years, gel in container in children aged ≥ 6 years [11].
      − Use neutral sodium fluoride 0.05% mouthwash daily for 3 weeks a month, 2–3 times a day after brushing teeth: use 15 mL, swish for 30 s and spit [62,65].
      − Use chlorhexidine 0.12% mouthwash (alcohol-free) daily for 1 week each month, 30 min after brushing teeth, at least twice a day: use 15 mL, swish for 30 s and spit [31,49,53,62,66].
      − In patients with poor oral hygiene and/or periodontal disease or mucositis, replace daily fluoride mouthwash with chlorhexidine 0.12% mouthwash (alcohol-free) daily (throughout the month, following the pattern indicated above) until oral tissue health improves. Fluoride toothpaste may be replaced by chlorhexidine toothpaste.
      − In patients with fungal infection, replace daily fluoride mouthwash with sodium bicarbonate 5% rinses or saline 0.9% rinses, daily during the entire month, 3 times a day after brushing teeth: use 15 mL for 30 s and spit. Continue until 1 week after fungal infection is resolved [6].
      − In patients aged < 6 years or who cannot control the swallowing reflex, impregnate gauze, cotton swabs, or sterile sponges with the mouthwash and gently clean the mucous membranes and gums after brushing [7,36,42].
  - Dietary advice: inform patient/parents of the importance of following a non-cariogenic diet: do not eat too many snacks, carbohydrate-rich supplements, or refined sugars; avoid foods with soda, sugary carbonated drinks, high doses of caffeine (coffee, cola drinks, etc.,) and fruit juices, the intake of irritant or spicy foods, foods with pasty consistency and food ingestion between meals [11,40]. Promote healthy snacks (e.g., carrot sticks). Limit intake of sucrose to a maximum of 3 intakes a day with free sugars intake up to 5% of daily energy [67,68]. Advise parents that some oral medications are sucrose rich.
  - Prevent trismus: perform daily exercises to stretch masticatory muscles (start before radiotherapy and continue afterward) [69].
  - Avoid or reduce radiation in healthy oral tissues: in patients requiring head and neck radiotherapy assess with the radiologist/oncologist the use of lead-coated stents, prostheses, shields, or protectors and techniques for maximum salivary gland preservation (three-dimensional coating or intensity-modulated radiotherapy), which prevent the effects of radiation when administered [7,11,18,53,61].
  - Educate patients/parents on the importance of optimal oral hygiene before, during, and after cancer treatment and inform them about the complications and possible short- and long-term effects of cancer treatments on the oral cavity and craniofacial complex [70].
4. Dental treatments
  - Order of priority of oral treatments:
    (a) They must be completed at least 7–10 days before starting chemo/radiotherapy. When this is not possible or is delayed, provisional restorations and non-acute dental treatments may be used [55,58,61].
    (b) Prioritise treatment of acute infections, extractions, periodontal treatments (scaling and root planing), and elimination of sources of irritation of oral tissues over fillings (use glass ionomer if permanent sealing is not possible and composite for fillings with perfect isolation), permanent tooth endodontics and replacement of defective restorations [8,9,11,71].
    (c) Caries with the risk of pulpal infection or pain should be treated first, and incipient or small lesions should be treated with fluoride and/or sealed until definitive treatment is possible [53].
  - Considerations of pulp treatment in temporary teeth:
    (a) In cases of pulpal involvement without periapical involvement or furcation, pulpal therapy (pulpotomy and pulpectomy) may be evaluated before cancer treatment (provided the prognosis is good and the haematological status permits) [12,46,53,59].
    (b) In case of furcation or periapical infection in immunosuppressed patients, choose extraction as the treatment (after previous antibiotic therapy for 1 week (the antibiotic of choice is penicillin and, in patients allergic to it, clindamycin) [53].
    (c) In teeth with previous pulp treatment, perform clinical reviews and periodic radiographic controls to assess signs of internal or radicular resorption or failure of treatment with periapical infection [53].
  - Endodontic considerations in permanent teeth:
    (a) Endodontic treatment may be performed in symptomatic non-vital teeth at least 7 days before cancer treatment. If this is not possible or definitive endodontics cannot be performed in a single session, dental extraction after previous antibiotic therapy for one week (the antibiotic of choice is penicillin and, in patients allergic to it, clindamycin) [9,11].
    (b) Treatment should be postponed in asymptomatic non-vital teeth until the haematological situation is stable [53].
    (c) In teeth with previous endodontics with radiolucent periapical lesions, it is important to determine their cause: if the tooth has no current signs or symptoms of infection, pulp retreatment or tooth extraction is not necessary [59].
  - Considerations of orthodontic devices and space maintainers:
    (a) Orthodontic appliances must be retired if they pose a risk of injury to the oral tissues and/or if there is poor oral hygiene and there is a moderate/severe risk of mucositis [53].
    (b) Fixed space maintainers, such as a band or crown and loop or lingual arch, which do not irritate the soft tissues, should be left in place in patients with good oral hygiene, and adjusted or removed otherwise [6].
    (c) Removable space maintainers may be maintained while the patient is instructed to clean the device routinely and daily with an antimicrobial solution that prevents the risk of infection associated with the device, and should be removed if they later cease to be tolerated by the patient or cause tissue irritation.
    (d) If the space maintainer cannot be removed, orthodontic wax or vinyl protectors should be used to prevent soft tissue trauma [53].
  - Periodontal treatment considerations:
    (a) Eliminate supragingival calculus using periodontal scaling (periodontal problems must be eliminated before starting chemotherapy, which stimulates myelosuppression and could worsen chronic periodontal situations) [6,64].
    (b) Perform periodontal treatment of subgingival calculus (scaling and root planing) if required prior to bisphosphonate therapy [72].
    (c) In teeth in which periodontal treatment has a poor prognosis (periodontal pockets > 5 mm), dental extraction is recommended.
    (d) In the case of pericoronitis in erupting teeth, excess gingival tissue should be excised (gingivectomy) when the haematological state allows.
    (e) If the patient requires periodontal treatment (scaling and root planing) or another invasive procedure but has previously taken bisphosphonates, there is a risk of osteonecrosis and the decision should be made together with the medical team, the patient, and parents [53].
  - Considerations before dental extraction:
    (a) Teeth with a poor prognosis should ideally be extracted 2 weeks before or, at least, 7–10 days before the start of cancer treatment: non-restorable teeth, root remains, teeth with periodontal pockets> 5 mm, symptomatic impacted teeth, teeth with acute associated infections, teeth with furcation injuries (furcation exposure), teeth with significant bone loss or mobile teeth [11,18,61].
    (b) Surgical procedures should be as atraumatic as possible. Prepare bleeding control measures (sutures, sterile compression gauze, microfibrillar collagen sponges, and local haemostatic agents such as topical thrombin) [6,61].
5. Other specific measures
  - Drink 2 litres of water/day to maintain proper hydration [40].
  - The lips should be well lubricated with lip protectors [53,58,61,64].
  - Avoid desiccating environments (such as excessive air conditioning and heating).
  - Avoid and combat stress (through regular physical exercise, yoga, etc.). Assess the control of anxiety and excessive stress, if present, with pharmacological measures if the medical-psychologist team considers it appropriate.
  - Stimulate remaining salivary gland function, if possible (chew sugar-free gum without strong flavours or consume sugar-free candy, suck tablets of a similar composition, chew food, taste stimuli such as paraffin). If there is no residual salivary function, counteract the negative consequences with protection and external lubrication (use of lip balms). Assess administration of salivary substitutes (artificial saliva) or sialagogues (pharmacological stimulants that should be administered with caution, taking contraindications into account: anethole trithione, pilocarpine, cevimeline, bethanecol, carbachol, pyridostigmine, neostigmine, and distigmine) [37,38,73,74,75,76].
  - Monitoring and periodic oral hygiene by the dentist (through individualised oral hygiene program, scaling and root planing every 6 months–1 year). Frequent dental visits for check-ups and maintenance to prevent and treat incipient lesions [10,12,60].

### 3.3. Phase 2: From the Initiation of Chemotherapy or Radiotherapy until 30–45 Days Post-Therapy

Phase II, which lasts from the initiation of chemotherapy or radiotherapy until 30–45 days post-therapy (period of immunosuppression and myelosuppression) has the following aims:

- To maintain optimal oral health during cancer treatment.
- To prevent and treat oral complications or side effects derived from cancer treatment.
- To strengthen patient/parent education on the importance of following optimal oral hygiene to minimise oral problems and the resulting discomfort during and after cancer treatment.

### 3.4. Systematic Measures

1. Review medical records:
  - Determine the current medical situation or underlying disease (stage and prognosis).
  - Establish a multidisciplinary treatment plan (depending on the current haematological/systemic status, current oral health situation, cancer treatment cycles).
  - Review the current haematological status (total neutrophil and platelet counts) and presence of permanent venous catheter.
2. Review oral history:
  - Review existing oral hygiene habits.
  - A new intraoral examination should be done one or two weeks after starting chemotherapy or radiotherapy, at which time typically secondary acute injuries begin: perform new odontogram and periodontogram.
  - New extraoral examination of head and neck.
3. Maintenance of preventive strategies:
  - Reinforce patient/parent education: remember that at this stage and in the long term, cancer treatment may give rise to complications in oral cavity and craniofacial complex.
  - Continue the general oral maintenance and prevention measures initiated in phase I:
    (a) Reinforce oral hygiene and provide motivation for correct brushing (*). (* indicates to follow the guidelines indicated in phase I), regardless of the haematological status. If there is oral pain or mucositis and a soft-medium hardness toothbrush is not tolerated, use an ultra-soft toothbrush or gauze until the soft-medium hardness brush is tolerated.
    (b) Continue using fluoride toothpaste when brushing (*).
    (c) Use of dental floss/floss (*).
    (d) Maintain the preventive applications of fluorinated preparations and antimicrobial products (in mouthwash, gel, or varnish):
      − Topical application of fluoride gel or varnish (*).
      − Rinse with sodium fluoride mouthwash (*).
      − Rinsing with chlorhexidine mouthwash (*).
      − Rinsing with sodium bicarbonate or saline solution (*).
      − Patients < 6 years of age or with an immature swallow reflex should apply the rinse by impregnating sterile gauze with the solution (*).
  - Maintain healthy dietary habits (*).
  - Continue with prevention of trismus: performing daily stretching exercises of the masticatory muscles (initiated before radiotherapy and continued post-therapy) [11,69].
4. Treatment of oral complications derived from cancer treatment:
  - Specific manifestations of the oral mucosa:
    (a) Mucositis (courses with erythema, erosion, ulceration, hyposialia, and xerostomia):
      − Nutritional support: Avoid acid, spicy, hard, hot, or irritating food. Parenteral nutrition if needed [71].
      − Oral decontamination: Alcohol-free rinses with chlorhexidine 0.12% or sodium bicarbonate 5% (*) [6,32,35,45,47,77].
      − Pain control [6,10,33,58]: cryotherapy (ice chips), saline 0.9% rinses, topical anaesthetics or mucous rinses containing anaesthetic: benzocaine (aerosol, gel), lidocaine 2% (viscose, ointment or aerosol), diphenhydramine and dyclonine hydrochloride 0.5 or 1.0% solution-Dyclonine). Non-opioid and opioid analgesics. Low-power laser therapy [15,26,30,45].
      − Administer allopurinol, leucovorin and nystatin [41,43,58,61].
      − Other drugs for the treatment of mucositis include mucosal coating drugs (Amphojel, kaopectate); hydroxypropyl methylcellulose film-forming drugs such as Zilactin; Gelclair (hyaluronic acid gel); amifostine; fibronectin; vitamin E, prostaglandin E2; growth factors such as recombinant human keratinocyte growth factor-1 and intravenous human fibroblast growth factor-20 (which significantly reduce the incidence of oral mucositis) and anti-inflammatory agents such as oral rinses with benzidine hydrochloride or topical antioxidants such as RK-0202 (n-acetylcysteine) that reduce the severity of oral mucositis [6,11,69,78].
      − Relief of oral dryness: (see next section on xerostomia).
      − Oral bleeding treatment: (see next section on oral bleeding).
      − Atrophy, burning, erosions, and ulcerations: hyaluronic acid gel.
    (b) Lichenoid reactions: controls, postpone biopsy.
    (c) Biogenic granuloma: controls, postpone biopsy.
  - Specific manifestations of the salivary glands:
    (a) Hyposialia and/or xerostomía [64,73,74,75,76]:
      − If there is residual salivary function: drink water frequently in small sips, chew gum without sugar or strong flavours or consume candies without sugar; suck tablets of similar composition, chew consistent foods, gustatory stimuli such as paraffin, assess the option of acupuncture.
      − If there is no residual salivary function: protection and external lubrication (use of lip balms). Assess the option of administering salivary substitutes (artificial saliva) or sialagogues (pharmacological stimulants of cholinergic action that should be administered with caution, taking into account their contraindications: anethole trithione, pilocarpine, cevimeline, bethanecol, carbachol, pyridostigmine, neostigmine and distigmine).
    (b) Sialadenitis: Antibiotic therapy, abundant fluid intake, analgesics, and drainage of pus (when haematological status permits) [61].
    (c) Mucocele: controls, postpone excision, and excisional biopsy.
  - Specific musculoskeletal manifestations
    (a) Less elasticity, reducing the range of mobility, and limiting mouth opening (e.g., scleroderma): daily stretching of masticatory muscles, muscle relaxants, and analgesics [69].
    (b) Osteonecrosis of the jaw due to bisphosphonates and osteoradionecrosis:
      Treatment to relieve pain, control soft tissues, control bone infection, and prevent and reduce the progression of bone necrosis: chlorhexidine 0.12% rinses + antibiotic therapy + analgesics + reviews; depending on the degree of jaw osteonecrosis, bone remodeling surgery, or extensive bone resection surgery may be required.
  - Temporomandibular disorders (e.g., trismus):
    Perform daily stretching exercises of the masticatory muscles (initiated before radiotherapy and continued post-therapy), place prosthesis (relaxation plate) to help reduce fibrosis and treat established trismus, assess the option of injections at trigger points, and prescribe analgesics and muscle relaxants [6,11,15,69,79].
  - Sensory disorders
    (a) Dysgeusia: Administer zinc supplements [64,69].
    (b) Neuropathies: Controls.
    (c) Dental hypersensitivity: Desensitising pastes.
  - Alterations in teeth and guns
    (a) Dental demineralisation and rampant caries: Application of remineralising agents and/or coating with glass ionomer (until definitive treatment is possible) [79].
    (b) Gingival hypertrophy and desquamative gingivitis: antimicrobial agents (chlorhexidine 0.12% rinses), antibiotic therapy.
    (c) Acute periodontal infections (symptomatic): Antimicrobial agents (chlorhexidine 0.12% rinses), antibiotic therapy [79].
    (d) Chronic pre-existing periodontal infections (asymptomatic): Antimicrobial agents (chlorhexidine 0.12% rinses) and controls.
  - Non-specific tissue manifestations:
    (a) Oral bleeding:
      − Local intraoral bleeding: compression by sterile gauzes, topical use of haemostatic agents such as gelatine sponges or topical thrombin, antifibrinolytic rinses [69].
      − Systemic bleeding: transfusions of platelets or aminocaproic acid, among others.
      − Avoid surgical procedures and local anaesthetic blocks [69].
    (b) Opportunistic infections:
      Monitor in the mouth because these infections do not have the same clinical appearance as normal ones. Perform oral cultures and/or biopsies of all suspicious lesions (when the haematological state permits).
      − Bacterial infections. Antibiotic depending on the microorganism [11,79].
      − Viral (herpes simplex type I): topical acyclovir [10,11,69,79,80].
      − Fungal (candidiasis): topical azoles (fluconazole) or polyenes (nystatin 4 times a day, 7–14 days) [10,11,44,69,79,80].
      − Severe infections → systemic therapy.
    (c) Secondary tumours (squamous cell carcinoma): Control and treatment according to medical assessment.
    (d) Post-transplant lymphoproliferative disorders: Control and treatment according to a medical assessment.
    (e) Abnormalities of dental development (hypoplasia, enamel discoloration or opacities, cessation of dental development, alteration of shape, number, and dental/root development). Control (postpone rehabilitation with definitive fillings, reconstructions, fixed prostheses (veneers, crowns, dental bridges), orthodontics).
    (f) Craniofacial alterations. Control (postpone orthopaedics, orthodontics, and orthognathic surgery).
5. Dental treatments:
  In this phase, only emergency in-hospital treatments should be carried out after a joint assessment with the cancer team of the most appropriate time for the treatment (in the period between cycles of cancer treatment, when the haematological status is more stable or using local or systemic haemostatic measures and antibiotic prophylaxis) [9,12].
6. Other specific measures:
  - Maintain correct hydration (*).
  - Keep lips well lubricated (*).
  - Avoid desiccating environments (*).
  - Avoid and combat stress (*).
  - Monitoring of oral hygiene and regular check-ups by the dentist (through individualised oral hygiene program, with scaling and root planning every 6 months–1 year). Frequent dental visits for revision and maintenance, and prevention and treatment of incipient lesions [10,12,60].

### 3.5. Phase 3: Begins after Cancer Treatment and May Last from 1–2 Years to the Whole Life

The aims of phase III (begins 30–45 days after cancer treatment and may last from 1–2 years to a lifetime) are:

- To maintain optimal oral health after cancer treatment.
- To treat oral complications or long-term oral side effects derived from cancer treatment.
- To strengthen patient/parent education on the importance of lifetime optimal oral hygiene.

### 3.6. Systematic Measures

1. Review of medical records (*)
2. Review of oral history (*)
3. Maintenance of preventive strategies:
  - Continue with patient/parent education (*).
  - Continue with general oral maintenance and prevention measures (*).
  - Maintain preventive applications of fluorinated preparations and antimicrobial products (mouthwash, gel, or varnish):
    (a) Topical application of fluorine gel (in container) or varnish (*).
    (b) Rinsing with sodium fluoride mouthwash (*).
    (c) Rinsing with chlorhexidine mouthwash (*).
    (d) Rinsing with sodium bicarbonate or saline solution (*).
  - Maintain healthy dietary habits (*).
  - Prevent/treat trismus: performing daily exercises to stretch the masticatory muscles (initiated before radiotherapy and continued post-therapy), place prosthesis (relaxation plate) to help reduce fibrosis and treat established trismus, assess the option of injecting trigger points, and prescribe analgesics and muscle relaxants.
4. Treatment of long-term oral complications derived from cancer treatment:
  - Specific manifestations of oral mucosa:
    (a) Mucositis (courses with erythema, erosion, ulceration, hyposialia, and xerostomia): (**) (** = Follow the guidelines indicated in phase II).
    (b) Atrophy, burning, erosions, and ulcerations: (**).
    (c) Lichenoid reactions: Controls, biopsy (1-year post-cancer treatment).
    (d) Pyogenic granuloma: Controls, biopsy (1-year post-cancer treatment).
  - Specific manifestations of salivary glands
    (a) Hyposialia and/or xerostomia: (**).
  - Musculoskeletal manifestations.
    (a) Less elasticity, reducing the range of mobility and limiting the mouth opening (e.g., scleroderma): (**).
    (b) Osteonecrosis of the jaw due to bisphosphonates and osteoradionecrosis (**).
    (c) Temporomandibular disorders (e.g., trismus) (**).
  - Secondary tumours (squamous cell carcinoma):
    Control and treatment according to medical assessment.
  - Post-transplant lymphoproliferative disorders:
    Control and treatment according to medical assessment.
  - Abnormalities in dental development (hypoplasia, enamel discoloration or opacities, cessation of dental development, alteration of shape, number, and dental/root development):
    Definitive filling and reconstructions can be made 1 year after the end of cancer treatment and fixed prostheses (veneers, crowns, dental bridges) and orthodontic treatments from 2 years post-treatment [27].
  - Craniofacial alterations
    Orthopaedics, orthodontics, and orthognathic surgery from 2 years post-treatment.
5. Dental treatments:
  - After bone marrow transplant, continue for one year without invasive dental procedures. Protocol of maintenance and oral prevention during the first year (only preventive non-invasive treatments: fluoride application, resin preventive restorations, pit and fissure sealants, coating with glass ionomer) [12].
  - After the first year: incorporate invasive dental treatments and the treatment of the long-term effects of cancer treatment (craniofacial alterations and dental anomalies) [10].
  - Orthodontic treatments can be started or continued after at least two disease-free years (two years of survival after cancer treatment) when there is less risk of relapse and immunosuppressive drugs are no longer required [6,81].
  - Reinforcement of the importance of correct oral hygiene to patients/parents.
6. Other specific measures:
  - Maintain correct hydration (*).
  - Keep the lips well lubricated (*).
  - Avoid desiccating environments (*).
  - Avoid and combat stress (*).
  - Monitor oral hygiene and periodic controls by the dentist (through individualised oral hygiene program, scaling and root planing every 6 months–1 year). Frequent dental visits (at short intervals every 3–6 months or less if necessary), review and maintenance to prevent and treat incipient lesions or when patient has trismus or xerostomia [10,12,60].

**Figure 1:**
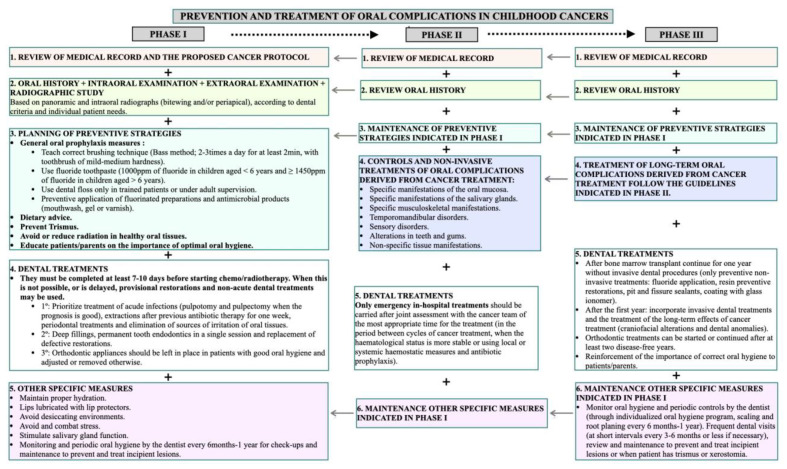
Flow chart regarding the patient management in phases 1-3 (pre, during, and post-treatment).

These results have been summarized in Figure 1 (Flow chart).

## 4. Conclusions

The use of standardised protocols based on prevention from early stages can avoid or minimise oral cancer complications and the side effects of cancer therapies, improving the quality of life of children.

Hospital trials will be necessary to apply the protocol for its subsequent clinical validation.

## Figures and Tables

**Table 1:** Oral complications in blood cancer patients and patients with bone marrow transplantation.

Category	Tissue	Oral Complication	Early Presentation	Late Presentation
Specific Tissue manifestations	Mucous membrane	Mucositis (approx. 100% children) [13,14,15,16].	+	+
		− Atrophy and burning. − Paleness (Anaemia). − Petechiae (22.6% children) [17]. − Ecchymosis (4.8% children) [17].	+	
		− Erosions and neutropenic ulcers (50% children) [17]. − Cracked lips (12.9% children) [17].	+	
		Lichenoid reactions, erythema, and ulcers (GVHD).		+
		Pyogenic granuloma	+	
	Salivary glands	Glandular hypofunction: Xerostomia (35.5% children) [17].	+	+
		Sialoadenitis	+	+
		Mucocele		+
	Musculoskeletal	Less elasticity, reducing the range of mobility and limiting the mouth opening (e.g., Scleroderma) (GVHD).		+
		Jaw osteonecrosis induced by therapy (osteoradionecrosis, or bisphosphonate-related osteonecrosis) [14].		+
		Temporomandibular disorders (eg. trismus). − ATM pain (6.5% children) [14,17].		+
	Sensory disoders	Dysgeusia/taste alteration [14].	+	+
		Neuropathies	+	+
		− Dental hypersensitivity − Oral pain (43.5% children) [17].		+
	Teeth and gums	− Dental mineralization and rampant caries [14,18]. − Dental abnormalities (short roots, tapering roots, enamel dysplasias, microdontia, tooth agenesis) [14,19].		
		− Gingival hypertrophy/hyperplasia [14].		+
		− Desquamative gingivitis (GVHD).		+
		− Acute periodontal infections (symptomatic). Gingivitis (38.7% children) [17]	+	+
		− Chronic pre-existing periodontal infections (asymptomatic)	+	+
Non specific Tissue manifestations		Oral bleeding (6–42% children) [8,14].	+	+
		Opportunistic infections: bacterial, viral and fungal. (herpes simplex infection: 9.7% children; candidiasis: 16.1% children) [14,15,17].	+	+
		Secondary tumours (e.g., squamous cell carcinoma). (3.2% children) [20].	+	+
		Post-transplant lymphoproliferative disorders (lymphadenopathies of head and neck: 11.2% children) [17].	+	+
		Abnormalities of dental development and craniofacial alterations in paediatric patients.	+	+

Note. GVHD: Graft versus host disease [Modification of table by Elad S, Raber-Durlacher J, Brennan MT, et al. 2015]. Early presentation: oral complications appear at the beginning of cancer treatment. Late presentation: oral complication appearing at the end of cancer treatment or after treatment.

## Supplementary Materials

The following supporting information can be downloaded at: https://www.mdpi.com/article/10.3390/children9040566/s1, File S1: Items selected for the review.
